# Metabolic Signature of Arrhythmogenic Cardiomyopathy

**DOI:** 10.3390/metabo11040195

**Published:** 2021-03-25

**Authors:** Chiara Volani, Johannes Rainer, Vinicius Veri Hernandes, Viviana Meraviglia, Peter Paul Pramstaller, Sigurður Vidir Smárason, Giulio Pompilio, Michela Casella, Elena Sommariva, Giuseppe Paglia, Alessandra Rossini

**Affiliations:** 1 Institute for Biomedicine, Eurac Research, Affiliated Institute of the University of Lübeck, Via Galvani 31, 39100 Bolzano, Italy; Johannes.Rainer@eurac.edu (J.R.); Vinicius.Veri@eurac.edu (V.V.H.); v.meraviglia@lumc.nl (V.M.); Peter.Pramstaller@eurac.edu (P.P.P.); runningman69@gmail.com (S.V.S.); Alessandra.Rossini@eurac.edu (A.R.); 2 Unit of Vascular Biology and Regenerative Medicine, Centro Cardiologico Monzino IRCCS, Via Parea 4, 20138 Milan, Italy; Giulio.Pompilio@cardiologicomonzino.it (G.P.); Elena.Sommariva@cardiologicomonzino.it (E.S.); 3 Department of Biomedical, Surgical and Dental Sciences, Università degli Studi di Milano, 20138 Milan, Italy; 4 Heart Rhythm Center, Centro Cardiologico Monzino IRCCS, 20138 Milan, Italy; michela.casella@ospedaliriuniti.marche.it; 5 Cardiology and Arrhythmology Clinic, University Hospital Ospedali Riuniti Umberto I-Lancisi-Salesi, 60126 Ancona, Italy; 6 Department of Clinical, Special and Dental Sciences, Marche Polytechnic University, 60126 Ancona, Italy; 7 School of Medicine and Surgery, Università degli Studi di Milano-Bicocca, 20854 Vedano al Lambro, Italy; giuseppe.paglia@unimib.it

**Keywords:** ACM, metabolomics, asymmetric dimethylarginine (ADMA), nitric oxide (NO), biocrates

## Abstract

Arrhythmogenic cardiomyopathy (ACM) is a genetic-based cardiac disease accompanied by severe ventricular arrhythmias and a progressive substitution of the myocardium with fibro-fatty tissue. ACM is often associated with sudden cardiac death. Due to the reduced penetrance and variable expressivity, the presence of a genetic defect is not conclusive, thus complicating the diagnosis of ACM. Recent studies on human induced pluripotent stem cells-derived cardiomyocytes (hiPSC-CMs) obtained from ACM individuals showed a dysregulated metabolic status, leading to the hypothesis that ACM pathology is characterized by an impairment in the energy metabolism. However, despite efforts having been made for the identification of ACM specific biomarkers, there is still a substantial lack of information regarding the whole metabolomic profile of ACM patients. The aim of the present study was to investigate the metabolic profiles of ACM patients compared to healthy controls (CTRLs). The targeted Biocrates AbsoluteIDQ^®^ p180 assay was used on plasma samples. Our analysis showed that ACM patients have a different metabolome compared to CTRLs, and that the pathways mainly affected include tryptophan metabolism, arginine and proline metabolism and beta oxidation of fatty acids. Altogether, our data indicated that the plasma metabolomes of arrhythmogenic cardiomyopathy patients show signs of endothelium damage and impaired nitric oxide (NO), fat, and energy metabolism.

## 1. Introduction

Metabolites are the intermediate or the end products of various cell processes [1] and closely reflect the phenotype, because they integrate individual’s genetic background, ageing and lifestyle [2]. Metabolites can be investigated individually or in combination within a comprehensive signature, being promising tools to understand the pathophysiological changes involved in the disease onset and progression [3,4].

Moreover, because many metabolites can be quantitatively detected in plasma, blood metabolomics has the potential to capture snapshots of the current biochemical pathways altered in different diseases [2] and be used for the discovery of new disease prediction and prognosis biomarkers [4]. 

The human metabolome is very complex in terms of chemical diversity, numerosity and different concentration ranges of the metabolites, therefore different methods have been developed for metabolite profiling. The approaches can be untargeted, mainly dedicated to biomarker discovery where metabolites are presented by means of relative quantification, or targeted, when specific classes of compounds are absolutely quantified with the benefit of increased sensitivity and selectivity [5]. Of note, one of the major limitations of the metabolomic approaches, especially of those based on liquid chromatography mass spectrometry (LC-MS) is still the lack of standardization [6]. Certified targeted LC-MS based assay platforms have become commercially available during the last few years (e.g., Biocrates, Metabolon) [7].

In the cardiovascular field, metabolomics has brought significant new insight not only into the metabolic changes occurring in cardiometabolic disorders [8], but also in myocardial infarction [9], cardiac reperfusion injury [10], heart failure [11], ischemic heart disease and atherosclerosis [12,13]. Recently, Alonso et al. described an association between serum glychocholenate sulfate (a secondary bile acid) and increased incidence of atrial fibrillation [14]. In addition, altered metabolomic pathways have been found in patients with either dilated (DCM) [15] or hypertrophic (HCM) [16] cardiomyopathy. 

Arrhythmogenic cardiomyopathy (ACM) is a genetic-based cardiac disease accompanied by severe ventricular arrhythmias, potentially leading to sudden death especially in young athletes [17]. Exercise may precipitate cardiac arrhythmias, presumably because of sympathetic over-activity [18]. The hallmark for ACM is the progressive substitution of the ventricular myocardium, mainly of the right ventricle, with fibro-fatty tissue [19]. The estimated prevalence is approximatively 1:5000 in the general population [20], although it appears more common in regions such as the north-east of Italy, where an intensive screening has been set-up [21].

ACM typically involves autosomal dominant transmission [22], the majority of known mutations occurring in genes responsible for mechanical connections, namely for the components of the cardiac desmosome [23] and, more in general, of the *area composita* and connexome [24,25,26].

Due to the reduced penetrance and variable expressivity [27], the presence of a genetic defect is not conclusive, and the broad phenotypic manifestation of the disease, ranging from ventricular arrhythmias to severe ventricular disfunction, makes it difficult to confirm the diagnosis of ACM [28].

Different studies have identified several potential biomarkers of ACM. Higher circulating levels of BIN1 (a regulator of calcium handling and cardiac contraction [29]), ST2 (interleukin-33 receptor, previously associated with cardiac remodeling, [30]), and GAL-3 (galectin-3, a marker of cardiac fibrosis [31,32]) have been described as possible predictors of ventricular arrhythmias, while reduced plasma expression of miR-320a has been proposed as a possible tool to discriminate ACM from idiopathic ventricular tachycardia [33].

More recently, the observation that human induced pluripotent stem cells-derived cardiomyocytes (iPSC-CMs) obtained from ACM individuals showed a dysregulated metabolic status characterized by excessive fatty acid oxidation (FAO) [34] led to the hypothesis that ACM patients can be characterized by markers of FAO-related metabolic alternations. In support of this hypothesis, plasma beta-hydroxybutyrrate (β-OHB) resulted higher in patients with ACM compared to healthy volunteers and was correlated with disease progression [35]. Along with that, the detrimental roles of a dysfunctional energetic and redox metabolism have been recognized as triggers for cardiomyocytes electrical instability and arrhythmogenesis [36].

Despite efforts having been made for the identification of ACM specific biomarkers, there is still a substantial lack of information regarding the whole metabolomic profile of ACM patients.

The aim of the present study was to define the metabolic profiles of ACM patient plasma compared to age- and gender-matched healthy controls. 

We used the targeted Biocrates AbsoluteIDQ^®^ p180 assay (*BIOCRATES* Life Sciences AG, Innsbruck, Austria) [37] to quantify 188 metabolites. This assay has already been used in several large-scale prospective cohort studies [38,39,40,41] and, due to its standardization, enables the inter-laboratory comparison of data obtained from different studies [42].

## 2. Results

A total of 36 patients were recruited at the Heart Rhythm Centre of Centro Cardiologico Monzino, if they met the ACM diagnostic criteria [43]. Most (88.89%) of the patients were male, with a mean age of 45 years. Two of them were overweight. Of them, 36.11% of them were athletes or very active people, defined according to COCIS (Comitato organizzativo cardiologico idoneità allo sport) scores [44] as when the subjects were undergoing three sessions/week of class C-D-E activities (or analogous). For controls, 27 healthy volunteers were enrolled with the criteria of match for all of these characteristics, which could influence the metabolic profile (Table 1).

Using a targeted approach, 188 metabolites and lipids were quantified; from these, 142 metabolites passed the pre-filtering criteria and were used in the subsequent analyses.

**Figure 1:**
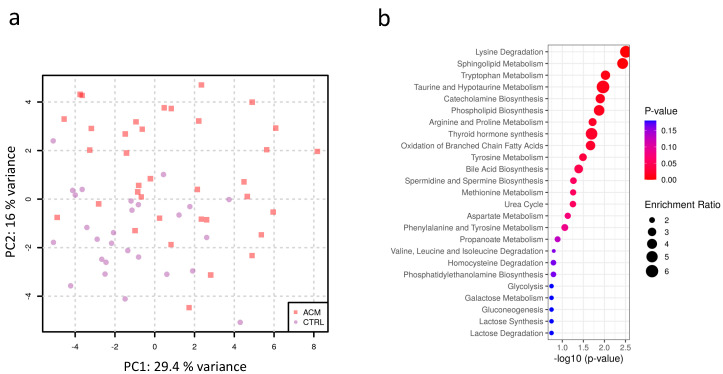
(**a**) Principal component analysis of plasma metabolites shows that ACM (red squares) clusters from CTRL (purple dots) samples (**b**) Enrichment analysis highlights the main pathways affected in ACM samples.

A principal component analysis (PCA) on these 142 analytes showed a separation of ACM plasma samples from control samples on PC1 (Figure 1a). Enrichment analysis (Figure 1b) revealed that several pathways were affected in ACM patients, including lysine degradation, sphingolipid metabolism, tryptophan metabolism, taurine metabolism, phospholipid biosynthesis, catecholamine biosynthesis, arginine metabolism, thyroid hormone synthesis, and the oxidation of branched chain fatty acids.

**Figure 2:**
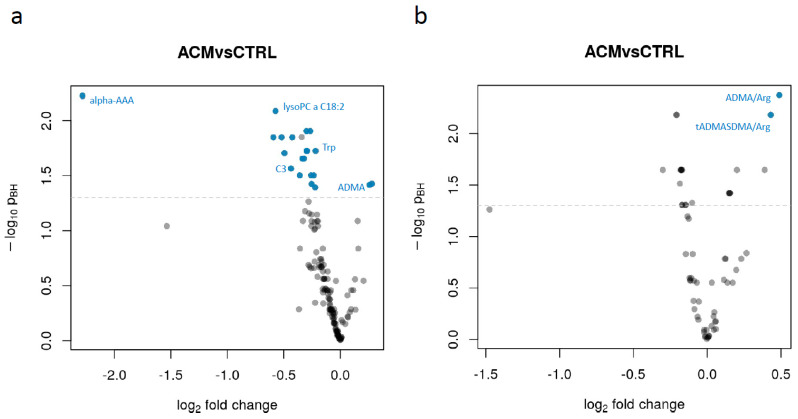
Volcano plots showing for each analyte the extent of differential abundance (log2 fold change) on the x-axis against its significance on the y-axis. (**a**) Results for individual metabolites, (**b**) results for metabolite sums and ratios. The blue dots indicate significant metabolites and metabolite sums/ratios. The name of some relevant metabolites is also fully reported. Alpha-AAA: alpha-aminoadipic acid; C3: carnitine C3; Trp: tryptophan; ADMA: asymmetric dimethylarginine; SDMA: symmetric dimethylarginine; Arg: arginine.

**Figure 3:**
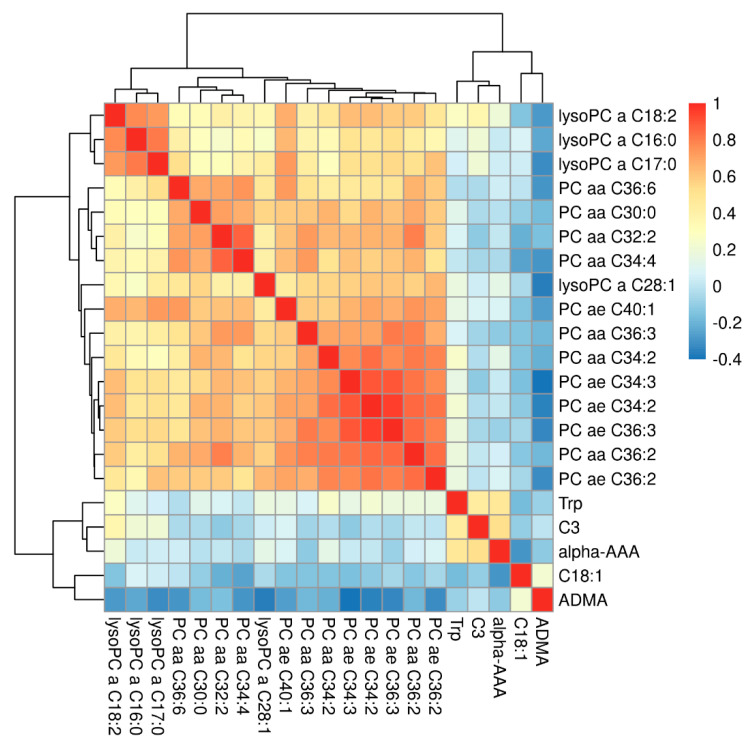
Correlations between significant metabolites.

We then performed a linear model-based differential abundance analysis to identify metabolites, as well as metabolite sums and ratios with significant differences between ACM and control samples. We did not find any metabolite to be significantly related to obesity (*N* = 3) nor to physical activity. Only creatinine was found to be significantly associated with sex, having an approximately 30% higher concentration in male participants compared to females which corresponds to the clinically used reference levels. Additional analyses relating metabolite abundances to severity of the disease (considering low left or right ventricular ejection fraction or presentation with ventricular tachycardia, VT) did also not yield significant findings (data not shown). In contrast, 21 metabolites and 2 analyte ratios/sums were found to have significant differences between ACM and control samples (Figure 2, Table 2). The overall metabolites and ratios assessed are listed in Supplementary Tables S1 and S2, respectively. The pairwise correlation between the significant metabolites is shown in Figure 3. As expected, higher correlations were present between metabolites of the same class.

**Figure 4:**
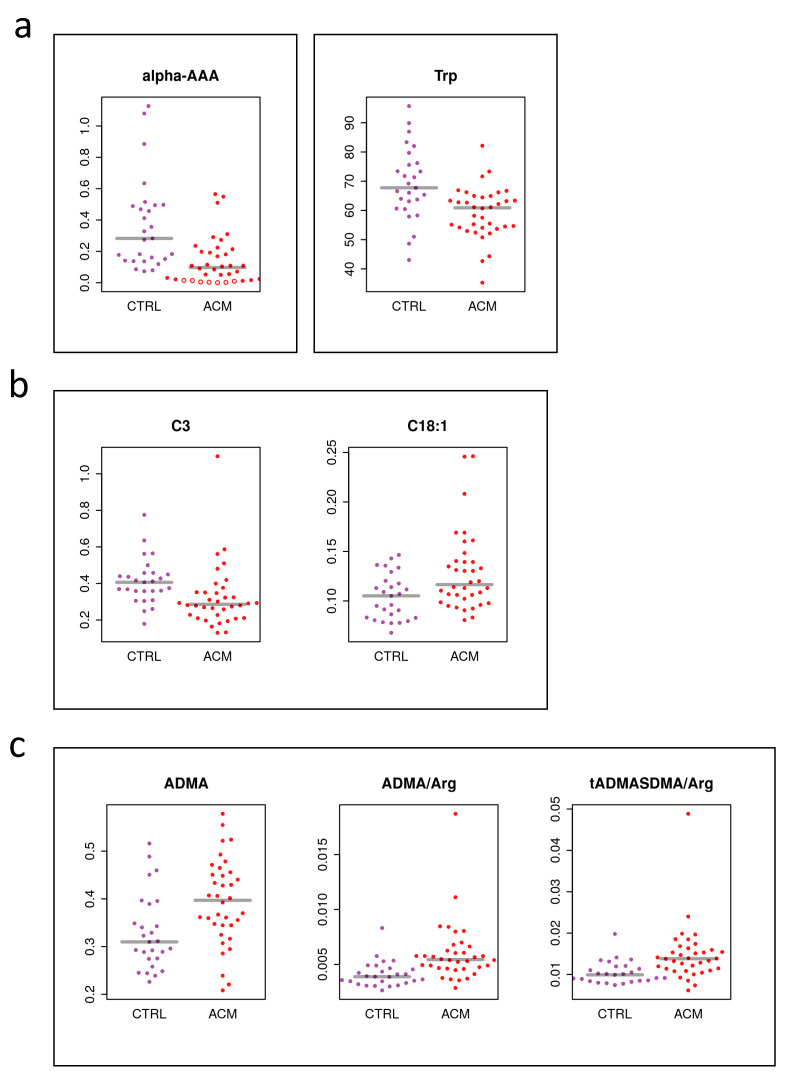
Individual analyte concentrations (adjusted for sex and batch) for significant metabolites (**a**,**b**) and metabolite ratios (**c**). CTRL (purple dots), ACM (red dots). Open circles indicate imputed values for original measurements being below the system’s detection limit. Alpha-AAA: alpha-aminoadipic acid; Trp: tryptophan; C3: carnitine C3; Arg: arginine; ADMA: asymmetric dimethylarginine.

**Figure 5:**
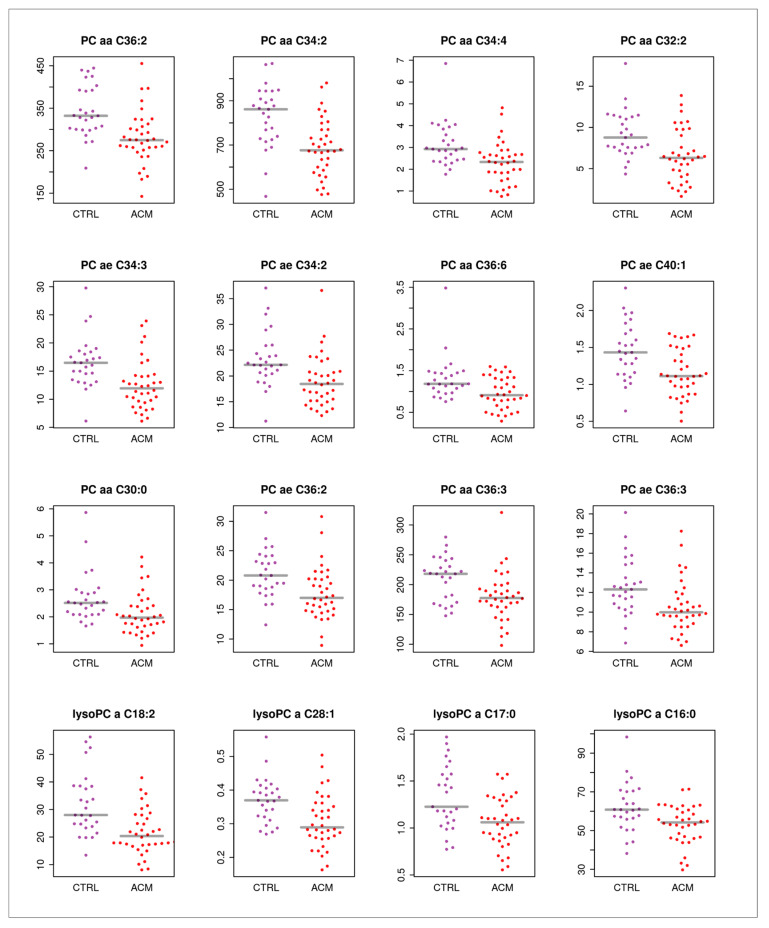
Individual analyte concentrations (adjusted for sex and batch) for glycerophospholipids with significant differences between ACM (red dots) and control samples (purple dots).

Key components of the affected pathways highlighted in Figure 1b were among the significant metabolites and ratios. This included α-aminoadipic acid (alpha-AAA, Figure 4a), which is generated in the body from the breakdown of lysine and was found to be downregulated in ACM patients compared to controls. Propionyl carnitine (C3), an acyl carnitine involved in beta-oxidation, was upregulated in ACM compared to controls (Figure 4b). Of interest, the ratios of asymmetric dimethylarginine (ADMA)/Arginine (Arg) and total asymmetric dimethylarginine and symmetric dimethylarginine (tADMASDMA)/Arg were increased in ACM patients, which resulted in elevated plasma levels of ADMA (Figure 4c). In addition, some glycerophospholipids were found to change significantly in ACM patients (Figure 5).

## 3. Discussion

Arrhythmogenic cardiomyopathy (ACM) is a genetic-based cardiac disease accompanied by severe ventricular arrhythmias and a progressive substitution of the myocardium with fibro-fatty tissue, which can lead to sudden death. Thus far, no single standard parameter defines ACM severity and progression, nor the genetic background or the phenotypic manifestation [45].

Contemporary human and cell culture studies have recognized an important role of the energetic metabolism [34,36] in the pathophysiology of ACM. In fact, along with the electro-mechanical dysfunction, ACM might be characterized by an altered metabolic status, that worsens the phenotype, and leads to tissue and systemic stress and damage.

Despite efforts having been made for the identification of ACM-specific biomarkers, there is still little known about the metabolomic profile of ACM patients. Thus, the current study aimed to investigate the metabolic profiles of ACM patient plasma compared to age-and gender-matched healthy controls. Even though it is known that the metabolic profile might depend on disease severity [46], our study was based on a mixed population characterized by different phenotypic manifestations. As such, trying to stratify metabolites according to disease severity (defined by left ventricle ejection fraction (LV EF), right ventricle ejection fraction (RV EF)) parameters or ventricular tachycardia (VT) at presentation) showed no significant association, most likely because of the low number of subjects per group after stratification (data not shown).

In accordance with the hypothesis of a modulated metabolism, our analysis showed that ACM patients have a different metabolome compared to CTRLs, and that the pathways mainly involved include lysine degradation, tryptophan metabolism, arginine and proline metabolism, and the beta oxidation of fatty acids.

Specifically, the ratio ADMA/Arg was found to be higher in ACM samples compared to controls, indicating an ongoing cardiovascular burden. This corresponded to increased plasma levels of ADMA in ACM. ADMA is produced by arginine methylation in proteins by the action of protein methyltransferases (PRMTs). Free ADMA is then released into the cytosol upon proteolysis and mainly excreted into the urine after being metabolized into citrulline and dimethylamine. However, a small percentage (around 20%) of dimethylarginines enter in the blood compartments and can be detected in plasma and serum [47,48].

ADMA is a surrogate marker of endothelial dysfunction, because it is an endogenous inhibitor of nitric oxide synthase competing with the natural substrate L-arginine thus decreasing nitric oxide (NO) synthesis [49]. NO is an endogenous vasodilator which inhibits the adhesion of inflammatory cells to vascular wall as well as the aggregation of platelets and proliferation of muscle cells. Therefore, increased ADMA levels and subsequent NO synthesis inhibition leads to vasoconstriction, reduced peripheral blood flow, and reduced cardiac output and it has been associated with hypertension, hyperlipidemia, and type II diabetes mellitus [12,50,51,52,53,54,55,56].

Moreover, higher levels of ADMA can predispose to the arrhythmic phenotype [57,58], as a result of a loss in the heart redox homeostasis [59].

Oxidative stress has been implicated in the pathogenesis of arrhythmic pathologies, such as atrial fibrillation [59]. There are multiple systems in the myocardium which contribute to redox homeostasis, and loss of homeostasis can result in oxidative stress. In particular, the crosstalk between reactive oxygen species (ROS) and nitric oxide synthase (NOS) could play an important role in regulating cardiomyocyte’s electro-mechanical function [60].

Along with the interplay between ROS and NO, the metabolism and beta oxidation of fatty acids could play an important role in the development and manifestation of ACM. The phenotypic manifestation of this pathology can in fact include fatty replacement in the heart; thus circulating free fatty acids can represent interesting signaling molecules to be investigated.

Among the free fatty acids, glycerophospholipids have recently received attention as potential biomarkers of cardiovascular disease. Phosphatidylcholines (PCs) and lysophosphatidylcholines (lysoPCs) are important members of the glycerophospholipid family [61]. PC levels in plasma have been correlated with aortic stiffness in CAD and PAD. Decreased serum levels of several individual PC and lysoPC species (e.g., PC aa C28:1, PC aa C30:0, PC aa C32:2, PC ae C30:0 and PC ae C34:2, lysoPC a C18:2) were observed for the patient groups in comparison to the healthy subjects. In addition, a considerable number of PCs and lysoPCs were inversely related to either cf-PWV, heart rate, asymmetric dimethylarginine (ADMA) or ADMA/arginine for patients with symptomatic atherosclerosis but not for the controls [61].

Plasma tryptophan (Trp) has been inversely associated with cardiovascular disease [62,63,64,65]. In addition, the involvement of Trp catabolism has been reported as one of the main mechanisms responsible for oxidative stress generation, and immune system activation [64,66]. Additionally, in our dataset, ACM patients showed lower plasma levels of trypotophan compared to controls, suggesting an increased breakdown of Trp, which can possibly lead to endothelial injury ref.

LysoPCs are bioactive lipids involved in monocyte recruitment, vascular smooth muscle cell proliferation, and endothelial dysfunction. These lysophospholipids are highly abundant in extracellular environments such as plasma, and interstitial fluids. 

An altered lipid metabolic signature was reported in cardiomyopathies and were indicated as potential new therapeutic options for the prevention and treatment of ischemic cardiomyopathy [67,68]. Accordingly, our data show that the plasma levels of several PCs and LyoPCs are decreased in ACM patients compared to controls. 

In addition, the literature reports an enhanced cardiovascular risk in the presence of impaired levels of circulating acylcarnitines [69,70]. Carnitines are small molecules that carry out a plethora of functions. Besides being involved in the transport of long-chain fatty acids from the cytosol into the mitochondria, carnitines play an important role in energy production, through the coordination of the activity of several enzymes of the Krebs cycle, beta oxidation, urea cycle and gluconeogenesis [71]. If their balance is maintained correctly, carnitines improve glucose tolerance, on the contrary this can promote several pathological consequences [71].

In our study, we found lower levels of carnitine C3 in ACM patients compared to controls. Carnitine C3, also called propionyl carnitine, is formed via carnitine acetyltransferase from propionyl-CoA, a product of methionine, threonine, valine, and isoleucine, as well as of odd-chain fatty acids. Carnitine C3 is highly specific for skeletal and cardiac muscle, because it carries the propionyl group and enhances the uptake of this agent by myocardial cells. These cells can use this metabolite as an anaplerotic substrate, thus providing energy in the absence of oxygen consumption [72]. Lower levels of C3 carnitine found in ACM patients can therefore indicate an energetic impairment.

Additionally, we observed lower levels of alpha-AAA. This metabolite is an intermediate of the lysine degradation metabolism. Studies indicate an association between alpha-aminoadipic acid and obesity, T2D development and CVD risk [73]. In particular, Gao et al described increased levels of alpha-AAA in unhealthy subjects compared to healthy ones [73]. Conversely, our data show that alpha-AAA is lower in ACM subjects compared to controls. In comparison with the studies reported above, most of the ACM subjects included in the study did not suffer either from obesity, or from T2D. Moreover, generally ACM subjects are athletes. Therefore, altered plasma levels of alpha-AAA in ACM might indicate an ongoing metabolic remodeling.

In accordance with this, Xu et al. [74] recently showed that the administration of 2-AAA protects mice from fat accumulation, by acting on their energetic expenditure system.

Altogether, our data indicate that arrhythmogenic cardiomyopathy patients are characterized by a different metabolome, pointing to endothelium damage and impaired NO, fat and energy metabolism.

These observations are consistent with the data reported so far on cardiovascular disease burden. The effect of this long-lasting metabolic status can promote arrhythmogenesis, endothelium damage and worsen the already impaired electro-mechanical function of the heart.

Our study showed that ACM patients manifest a peripheral metabolism characterized by a status of redox imbalance, impaired fatty acid metabolism and NO synthesis. As such, metabolic phenotyping could offer the possibility to be used to monitor the health of subjects at risk of developing arrhythmias, such as athletes.

While endothelium damage, impaired NO, fat and energy metabolism can be considered the hallmark for all the non-ischemic heart failure and/or cardiovascular burden generally [51,66,75,76], alteration of lysine degradation might represent a specific pathway involved in ACM. However, further studies are needed to confirm these findings. Moreover, even if biomarker discovery was not the main scope of this study, we foresee that plasma level of alpha-AAA might be a good candidate for a better evaluation focused on biomarker discovery on ACM.

## 4. Materials and Methods

### 4.1. Ethical Statement

This study complied with the Declaration of Helsinki and was approved by the Ethics Committees of the Centro Cardiologico Monzino (6 June 2012), and of the Azienda Sanitaria dell’Alto Adige (Nr. 1/2014, 13 March 2014). Written informed consent was obtained from all participants.

### 4.2. Participants

A total of 63 subjects were enrolled in one recruiting centre (Centro Cardiologico Monzino): 27 healthy controls (CTRL) and 36 patients affected by ACM, as per the task force criteria [43]. Controls were individuals apparently in good health with no history of cardiovascular disease and were matched by sex and age to ACM patients. Characteristics of the enrolled subject are reported in Table 1. Among the ACM and the CTRL groups, no significant differences were found in terms of sex, age, obesity and athletic lifestyle.

### 4.3. Plasma Preparation

Blood samples (5 mL) were collected in EDTA coated tubes and centrifuged at 1500× *g* for 15 min. Supernatants were collected, centrifuged again at 16,000× *g* for 15 min to obtain cell- and platelet-free plasma, and stored at −80 °C as 400 µL aliquots until usage.

### 4.4. Metabolite Quantification

Targeted metabolomics analysis of plasma samples was performed using the Biocrates AbsoluteIDQ^®^ p180 kit (*BIOCRATES* Life Sciences AG, Innsbruck, Austria) with an ultra-high performance liquid chromatography (UHPLC) (Agilent 1290, Agilent Technologies, Santa Clara, CA, USA) coupled to a Q-Trap mass spectrometer (MS) (QTRAP 6500, Sciex, Foster City, CA, USA). Sample preparation and analysis were performed according to the manufacturer’s protocol. In brief, the sample processing procedure was performed on a single sample and utilized a 96-well plate design, where both sample derivatization and analyte extraction were performed. The kit required 10 μL of sample and provided human plasma-based quality controls in 3 concentration levels (low, medium, high) which could be used for quality control purposes and batch normalization. Once processed, each sample was subjected to two separate MS-based analytical runs. From the 188 metabolites that could be measured using the kit, 42 were quantified by UHPLC-MS/MS (namely 21 amino acids and 21 biogenic amines), with the use of external calibration standards in seven different concentrations and isotope labelled internal standards. Moreover, 146 metabolites were analyzed using flow-injection analysis (FIA-MS/MS) (more specifically 40 acylcarnitines, 90 glycerophospholipids, 15 sphingolipids and 1 sum of hexoses), in a semi-quantitative methodology, using one-point internal standard calibration with representative internal standards Furthermore, it is important to highlight that several lipids analyzed in the present kit represent the total concentrations of possible isobars and structural isomers. In general, lipid annotations are represented by C *x*:*y*, where *x* indicates the number of carbons and *y* indicates the number of double bonds. Glycerophospholipids are further differentiated according to the presence of ester (a) and ether (e) bonds in the glycerol moiety. Double letters (aa = diacyl, ae = acyl–alkyl) indicate that two glycerol positions are bound to a fatty acid residue, while a single letter (a = acyl or e = alkyl) indicates a bond with only one fatty acid residue.

The calculations of metabolite concentrations were performed using the MetIDQ™ (BIOCRATES Life Sciences AG, Innsbruck, Austria) software package, which is an integral part of the AbsoluteIDQ kit. The concentrations of metabolites were calculated in μM (10^−6^ mol/L).

### 4.5. Statistical Data Analysis

Data analysis was performed in R (version 4.0.2). The dataset of 188 analytes was first prefiltered keeping only metabolites with at least 30% of measurements in one of the two study groups above the lower level of detection (LOD; defined by Biocrates) and a relative standard deviation (RSD) <= 20% for two out of three QC sample types (i.e., the pool of all samples in the present study as well as the kit-internal QC sample types 00p180_QC1 and 00p180_QCs). In total 142 analytes fulfilled the above criteria and were therefore used for the further analysis. The few missing values in the dataset were replaced by a random value smaller than half of the analyte’s smallest reported concentration. Analyte sums and ratios were based on the information/definition from Biocrates and were calculated from the individual analytes’ concentrations. Raw p-values for significant differences in abundances were estimated using the limma package [77], fitting a linear model adjusting for the participants’ sex, obesity and the batch in which samples were collected. To evaluate the impact of physical activity of participants on their metabolite levels we fitted a linear model including a categorical variable for physical activity. The analyses to identify metabolites related to disease severity were performed on ACM patient data only by fitting separate linear models with a categorical variable for disease severity. In all models we adjusted for sex, age, batch and obesity. For disease severity we considered ventricular tachycardia at presentation (VT; n_yes_ = 13, n_no_ = 23), low right ventricular ejection fraction (RVEF < 45; n_low_ = 13, n_normal_ = 15, n_unknown_ = 8) or low left ventricular ejection fraction (LVEF < 50; n_low_ = 6, n_normal_ = 21, n_unknown_ = 9). Raw p-values were adjusted for multiple hypothesis testing with the method from Benjamini and Hochberg. Differential abundance analysis was also performed for analyte ratios and sums (as defined by Biocrates) using the same settings. Analytes were considered significant if they had an adjusted p-value smaller than 0.05 (representing a 5% false discovery rate), also with in addition requiring, for individual metabolites, a difference in concentrations being at least twice as large as the analyte’s RSD in QC samples, or a difference being larger than 30% for analyte sums and ratios.

For the enrichment analysis, log transformed data were mean centered and divided by the standard deviation of each variable. This analysis was based on Global Test using a small molecule pathway database (SPMPD) [78] and was performed using MetaboAnalyst [79]. Principal component analysis was performed on log2 transformed mean centered abundances.

## 5. Conclusions

Our study was aimed to investigate the metabolic profiles of ACM patient plasma compared to age-and gender-matched healthy controls. Altogether our data show that the plasma metabolomes of arrhythmogenic cardiomyopathy patients suggest signs of endothelium damage, impaired NO, fat and energy metabolism. Additional studies are required to replicate these findings and increase statistical power for novel discoveries. Additionally, the present study was limited to a set of 188 metabolites and more complete approaches might be required to discover changes in other metabolites and metabolite classes not detected by the methodology used in the present study. Although we tried to adjust for the most obvious potential confounding factors, we cannot exclude the analysis not being biased by the presence of other (hidden) confounding variables (such as diet). 

## Figures and Tables

**Table 1:** Demographic and clinical characteristics of enrolled subjects. ACM, arrhythmogenic cardiomyopathy; RV, right ventricle; LV, left ventricle; EF, ejection fraction; VT, ventricular tachycardia. Data are reported as percentages (%) or mean ± standard deviation (SD).

	ACM Patients (*N* = 36)	CTRL (*N* = 27)	*p* Value (Test)
Male sex (%; *n*)	88.89% (*n* = 32/36)	74.07% (*n* = 20/27)	0.18 (Fisher’s exact test)
Age (mean ± SD)	45.31 ± 13.29	46.04 ± 14.19	0.83 (Student *t*-test)
Obesity (%; *n*)	5.56% (*n* = 2/36)	0.04% (*n* = 1/27)	1.00 (Fisher’s exact test)
Athletic lifestyle (%; *n*)	36.11% (*n* = 13/36)	36.36% (*n* = 8/22)	1.00 (Fisher’s exact test)
RV EF % (mean ± SD)	44.43% ± 7.79	ND	
LV EF % (mean ± SD)	54.63% ± 13.64	ND	
VT at presentation (%; *n*)	63.89% (*n* = 23/36)	ND	

**Table 2:** List of the significant metabolites and ratios. The table shows the analyte name, the log2 fold change and the *p*-value adjusted for multiple hypothesis testing. Analytes were sorted by the difference in abundance.

Name	Log_2_ Fold Change	*p*_BH_
alpha-AAA	−2.2817718	0.00599521
PC aa C32:2	−0.5926965	0.01416184
lysoPC a C18:2	−0.5736359	0.00817355
PC aa C34:4	−0.519949	0.01416184
PC aa C36:6	−0.4945815	0.01972115
C3	−0.436115	0.02721582
PC ae C34:3	−0.4253616	0.01416184
PC aa C30:0	−0.3589317	0.03137787
PC ae C40:1	−0.3355771	0.02210991
lysoPC a C17:0	−0.322115	0.02210991
PC aa C36:2	−0.2985986	0.01241476
PC ae C34:2	−0.297975	0.01884033
lysoPC a C28:1	−0.2948432	0.01884033
PC aa C34:2	−0.2661214	0.01241476
PC ae C36:2	−0.2586741	0.03137787
PC ae C36:3	-0.2546263	0.03763856
PC aa C36:3	−0.2350526	0.03137787
lysoPC a C16:0	−0.2223977	0.04041611
Trp	−0.2181965	0.01884033
ADMA	0.25872497	0.03833323
C18:1	0.28073695	0.03750482
ADMA/Arg	0.48864835	0.00423649
tADMASDMA/Arg	0.43152368	0.0065969

## Data Availability

The data presented in this study are available in Supplementary Materials.

## Supplementary Materials

The following are available online at https://www.mdpi.com/article/10.3390/metabo11040195/s1, The supplementary material includes the complete table of metabolites (Table S1) and ratios (Table S2) assessed during the analysis. The significant metabolites and ratios were highlighted in yellow.

## References

[1] Guijas C., Montenegro-Burke J.R., Warth B., Spilker M.E., Siuzdak G. (2018). Metabolomics activity screening for identifying metabolites that modulate phenotype. Nat. Biotechnol..

[2] Nicholson J.K. (2006). Global systems biology, personalized medicine and molecular epidemiology. Mol. Syst. Biol..

[3] Donatti A., Canto A.M., Godoi A.B., Da Rosa D.C., Lopes-Cendes I. (2020). Circulating Metabolites as Potential Biomarkers for Neurological Disorders—Metabolites in Neurological Disorders. Metabolites.

[4] Bujak R., Struck-Lewicka W., Markuszewski M.J., Kaliszan R. (2015). Metabolomics for laboratory diagnostics. J. Pharm. Biomed. Anal..

[5] McGarrah R.W., Crown S.B., Zhang G.-F., Shah S.H., Newgard C.B. (2018). Cardiovascular Metabolomics. Circ. Res..

[6] Dias D.A., Koal T. (2016). Progress in Metabolomics Standardisation and its Significance in Future Clinical Laboratory Medicine. EJIFCC.

[7] Monnerie S., Comte B., Ziegler D., Morais J.A., Pujos-Guillot E., Gaudreau P. (2020). Metabolomic and Lipidomic Signatures of Metabolic Syndrome and its Physiological Components in Adults: A Systematic Review. Sci. Rep..

[8] Rankin N.J., Preiss D., Welsh P., Sattar N. (2016). Applying metabolomics to cardiometabolic intervention studies and trials: Past experiences and a roadmap for the future: Table 1. Int. J. Epidemiol..

[9] Zhu M., Han Y., Zhang Y., Zhang S., Wei C., Cong Z., Du W. (2018). Metabolomics Study of the Biochemical Changes in the Plasma of Myocardial Infarction Patients. Front. Physiol..

[10] Surendran A., Aliani M., Ravandi A. (2019). Metabolomic characterization of myocardial ischemia-reperfusion injury in ST-segment elevation myocardial infarction patients undergoing percutaneous coronary intervention. Sci. Rep..

[11] Marcinkiewicz-Siemion M., Ciborowski M., Kretowski A., Musial W., Kaminski K. (2016). Metabolomics—A wide-open door to personalized treatment in chronic heart failure?. Int. J. Cardiol..

[12] Shimada Y.J., Batra J., Kochav S.M., Patel P., Jung J., Maurer M.S., Hasegawa K., Reilly M.P., Fifer M.A. (2020). Difference in Metabolomic Response to Exercise between Patients with and without Hypertrophic Cardiomyopathy. J. Cardiovasc. Transl. Res..

[13] Del Greco M F., Foco L., Teumer A., Verweij N., Paglia G., Meraviglia V., Melotti R., Vukovic V., Rauhe W., Joshi P.K. (2019). Lipidomics, Atrial Conduction, and Body Mass Index. Circ. Genom. Precis. Med..

[14] Alonso A., Yu B., Sun Y.V., Chen L.Y., Loehr L.R., O’Neal W.T., Soliman E.Z., Boerwinkle E. (2019). Serum Metabolomics and Incidence of Atrial Fibrillation (from the Atherosclerosis Risk in Communities Study). Am. J. Cardiol..

[15] Alexander D., Lombardi R., Rodriguez G., Mitchell M.M., Marian A.J. (2010). Metabolomic distinction and insights into the pathogenesis of human primary dilated cardiomyopathy. Eur. J. Clin. Investig..

[16] Jorgenrud B., Jalanko M., Helio T., Jaaskelainen P., Laine M., Hilvo M., Nieminen M.S., Laakso M., Hyotylainen T., Oresic M. (2015). The Metabolome in Finnish Carriers of the MYBPC3-Q1061X Mutation for Hypertrophic Cardiomyopathy. PLoS ONE.

[17] Corrado D., Basso C., Schiavon M., Thiene G. (1998). Screening for Hypertrophic Cardiomyopathy in Young Athletes. N. Engl. J. Med..

[18] Riele A.S.T., Ajijola O.A., Shivkumar K., Tandri H. (2016). Role of Bilateral Sympathectomy in the Treatment of Refractory Ventricular Arrhythmias in Arrhythmogenic Right Ventricular Dysplasia/Cardiomyopathy. Circ. Arrhythmia Electrophysiol..

[19] Corrado D., Link M.S., Calkins H. (2017). Arrhythmogenic Right Ventricular Cardiomyopathy. N. Engl. J. Med..

[20] Peters S., Trümmel M., Meyners W. (2004). Prevalence of right ventricular dysplasia-cardiomyopathy in a non-referral hospital. Int. J. Cardiol..

[21] Corrado D., Basso C., Pavei A., Michieli P., Schiavon M., Thiene G. (2006). Trends in Sudden Cardiovascular Death in Young Competitive Athletes After Implementation of a Preparticipation Screening Program. JAMA.

[22] Marcus F.I., Edson S., Towbin J.A. (2013). Genetics of Arrhythmogenic Right Ventricular Cardiomyopathy. J. Am. Coll. Cardiol..

[23] Delmar M., McKenna W.J. (2010). The Cardiac Desmosome and Arrhythmogenic Cardiomyopathies. Circ. Res..

[24] De Bortoli M., Postma A.V., Poloni G., Calore M., Minervini G., Mazzotti E., Rigato I., Ebert M., Lorenzon A., Vazza G. (2018). Whole-Exome Sequencing Identifies Pathogenic Variants in TJP1 Gene Associated With Arrhythmogenic Cardiomyopathy. Circ. Genom. Precis. Med..

[25] Mayosi B.M., Fish M., Shaboodien G., Mastantuono E., Kraus S., Wieland T., Kotta M.-C., Chin A., Laing N., Ntusi N.B. (2017). Identification of Cadherin 2 (CDH2) Mutations in Arrhythmogenic Right Ventricular Cardiomyopathy. Circ. Cardiovasc. Genet..

[26] Van Hengel J., Calore M., Bauce B., Dazzo E., Mazzotti E., De Bortoli M., Lorenzon A., Li Mura I.E., Beffagna G., Rigato I. (2013). Mutations in the area composita protein alphaT-catenin are associated with arrhythmogenic right ventricular cardiomyopathy. Eur. Heart J..

[27] Dalal D., James C., Devanagondi R., Tichnell C., Tucker A., Prakasa K., Spevak P.J., Bluemke D.A., Abraham T., Russell S.D. (2006). Penetrance of mutations in plakophilin-2 among families with arrhythmogenic right ventricular dysplasia/cardiomyopathy. J. Am. Coll. Cardiol..

[28] Corrado D., Van Tintelen P.J., McKenna W.J., Hauer R.N.W., Anastastakis A., Asimaki A., Basso C., Bauce B., Brunckhorst C., Bucciarelli-Ducci C. (2019). Arrhythmogenic right ventricular cardiomyopathy: Evaluation of the current diagnostic criteria and differential diagnosis. Eur. Heart J..

[29] Hong T.-T., Cogswell R., James C.A., Kang G., Pullinger C.R., Malloy M.J., Kane J.P., Wojciak J., Calkins H., Scheinman M.M. (2012). Plasma BIN1 correlates with heart failure and predicts arrhythmia in patients with arrhythmogenic right ventricular cardiomyopathy. Heart Rhythm..

[30] Broch K., Leren I.S., Saberniak J., Ueland T., Edvardsen T., Gullestad L., Haugaa K.H. (2017). Soluble ST2 is associated with disease severity in arrhythmogenic right ventricular cardiomyopathy. Biomarkers.

[31] Oz F., Onur I., Elitok A., Ademoglu E., Altun I., Bilge A.K., Adalet K. (2017). Galectin-3 correlates with arrhythmogenic right ventricular cardiomyopathy and predicts the risk of ventricular -arrhythmias in patients with implantable defibrillators. Acta Cardiol..

[32] Stadiotti I., Pompilio G., Maione A.S., Pilato C.A., D’Alessandra Y., Sommariva E. (2019). Arrhythmogenic cardiomyopathy: What blood can reveal?. Heart Rhythm..

[33] Sommariva E., D’Alessandra Y., Farina F.M., Casella M., Cattaneo F., Catto V., Chiesa M., Stadiotti I., Brambilla S., Russo A.D. (2017). MiR-320a as a Potential Novel Circulating Biomarker of Arrhythmogenic CardioMyopathy. Sci. Rep..

[34] Kim C., Wong J., Wen J., Wang S., Wang C., Spiering S., Kan N.G., Forcales S., Puri P.L., Leone T.C. (2013). Studying arrhythmogenic right ventricular dysplasia with patient-specific iPSCs. Nature.

[35] Song J.-P., Chen L., Chen X., Ren J., Zhang N.-N., Tirasawasdichai T., Hu Z.-L., Hua W., Hu Y.-R., Tang H.-R. (2020). Elevated plasma β-hydroxybutyrate predicts adverse outcomes and disease progression in patients with arrhythmogenic cardiomyopathy. Sci. Transl. Med..

[36] Barth A.S., Tomaselli G.F. (2009). Cardiac Metabolism and Arrhythmias. Circ. Arrhythmia Electrophysiol..

[37] Paglia G., Del Greco F.M., Sigurdsson B.B., Rainer J., Volani C., Hicks A.A., Pramstaller P.P., Smarason S.V., Del Greco M.F. (2018). Influence of collection tubes during quantitative targeted metabolomics studies in human blood samples. Clin. Chim. Acta.

[38] Floegel A., Kühn T., Sookthai D., Johnson T., Prehn C., Rolle-Kampczyk U., Otto W., Weikert C., Illig T., Von Bergen M. (2018). Serum metabolites and risk of myocardial infarction and ischemic stroke: A targeted metabolomic approach in two German prospective cohorts. Eur. J. Epidemiol..

[39] Floegel A., Stefan N., Yu Z., Mühlenbruch K., Drogan D., Joost H.-G., Fritsche A., Häring H.-U., De Angelis M.H., Peters A. (2012). Identification of Serum Metabolites Associated With Risk of Type 2 Diabetes Using a Targeted Metabolomic Approach. Diabetes.

[40] Kühn T., Floegel A., Sookthai D., Johnson T., Rolle-Kampczyk U., Otto W., Von Bergen M., Boeing H., Kaaks R. (2016). Higher plasma levels of lysophosphatidylcholine 18:0 are related to a lower risk of common cancers in a prospective metabolomics study. BMC Med..

[41] Suhre K., Meisinger C., Doring A., Altmaier E., Belcredi P., Gieger C., Chang D., Milburn M.V., Gall W.E., Weinberger K.M. (2010). Metabolic footprint of diabetes: A multiplatform metabolomics study in an epidemiological setting. PLoS ONE.

[42] Siskos A.P., Jain P., Romisch-Margl W., Bennett M., Achaintre D., Asad Y., Marney L., Richardson L., Koulman A., Griffin J.L. (2017). Interlaboratory Reproducibility of a Targeted Metabolomics Platform for Analysis of Human Serum and Plasma. Anal. Chem..

[43] Marcus F.I., McKenna W.J., Sherrill D., Basso C., Bauce B., Bluemke D.A., Calkins H., Corrado D., Cox M.G., Daubert J.P. (2010). Diagnosis of arrhythmogenic right ventricular cardiomyopathy/dysplasia: Proposed modification of the Task Force Criteria. Eur. Heart J..

[44] COMPOSIZIONE COMITATO COCIS (2017). Protocolli Cardiologici per il Giudizio di Idoneità allo Sport Agonistico Casa.

[45] Towbin J.A., McKenna W.J., Abrams D.J., Ackerman M.J., Calkins H., Darrieux F.C.C., Daubert J.P., de Chillou C., DePasquale E.C., Desai M.Y. (2019). 2019 HRS expert consensus statement on evaluation, risk stratification, and management of arrhythmogenic cardiomyopathy: Executive summary. Heart Rhythm..

[46] Verdonschot J.A.J., Wang P., Van Bilsen M., Hazebroek M.R., Merken J.J., Vanhoutte E.K., Henkens M., Van Den Wijngaard A., Glatz J.F.C., Krapels I.P.C. (2020). Metabolic Profiling Associates with Disease Severity in Nonischemic Dilated Cardiomyopathy. J. Card. Fail.

[47] D’Apolito O., Paglia G., Tricarico F., Garofalo D., Pilotti A., Lamacchia O., Cignarelli M., Corso G. (2008). Development and validation of a fast quantitative method for plasma dimethylarginines analysis using liquid chromatography-tandem mass spectrometry. Clin. Biochem..

[48] Paglia G., D’Apolito O., Tricarico F., Garofalo D., Corso G. (2008). Evaluation of mobile phase, ion pairing, and temperature influence on an HILIC-MS/MS method for L-arginine and its dimethylated derivatives detection. J. Sep. Sci..

[49] Vallance P., Leone A., Calver A., Collier J., Moncada S. (1992). Accumulation of an endogenous inhibitor of nitric oxide synthesis in chronic renal failure. Lancet.

[50] Chu R., Yu D., Chu J., Lin M., Yu H. (2018). Prognostic efficacy of circulating asymmetric dimethylarginine in patients with peripheral arterial disease: A meta-analysis of prospective cohort studies. Vascular.

[51] Schulze F., Lenzen H., Hanefeld C., Bartling A., Osterziel K.J., Goudeva L., Schmidt-Lucke C., Kusus M., Maas R., Schwedhelm E. (2006). Asymmetric dimethylarginine is an independent risk factor for coronary heart disease: Results from the multicenter Coronary Artery Risk Determination investigating the Influence of ADMA Concentration (CARDIAC) study. Am. Heart J..

[52] Cavusoglu E., Ruwende C., Chopra V., Yanamadala S., Eng C., Pinsky D.J., Marmur J.D. (2009). Relationship of baseline plasma ADMA levels to cardiovascular outcomes at 2 years in men with acute coronary syndrome referred for coronary angiography. Coron. Artery. Dis..

[53] Krempl T.K., Maas R., Sydow K., Meinertz T., Böger R.H., Kähler J. (2005). Elevation of asymmetric dimethylarginine in patients with unstable angina and recurrent cardiovascular events. Eur. Heart J..

[54] Cavusoglu E., Ruwende C., Chopra V., Poludasu S., Yanamadala S., Frishman W.H., Eng C., Pinsky D.J., Marmur J.D. (2010). Relation of baseline plasma ADMA levels to cardiovascular morbidity and mortality at two years in men with diabetes mellitus referred for coronary angiography. Atherosclerosis.

[55] Abedini S., Meinitzer A., Holme I., März W., Weihrauch G., Fellstrøm B., Jardine A., Holdaas H. (2010). Asymmetrical dimethylarginine is associated with renal and cardiovascular outcomes and all-cause mortality in renal transplant recipients. Kidney Int..

[56] Valkonen V.P., Päivä H., Salonen J.T., Lakka T.A., Lehtimäki T., Laakso J., Laaksonen R. (2001). Risk of acute coronary events and serum concentration of asymmetrical dimethylarginine. Lancet.

[57] Burger A.L., Stojkovic S., Diedrich A., Demyanets S., Wojta J., Pezawas T. (2020). Elevated plasma levels of asymmetric dimethylarginine and the risk for arrhythmic death in ischemic and non-ischemic, dilated cardiomyopathy—A prospective, controlled long-term study. Clin. Biochem..

[58] Horowitz J.D., De Caterina R., Heresztyn T., Alexander J.H., Andersson U., Lopes R.D., Steg P.G., Hylek E.M., Mohan P., Hanna M. (2018). Asymmetric and Symmetric Dimethylarginine Predict Outcomes in Patients With Atrial Fibrillation: An ARISTOTLE Substudy. J. Am. Coll. Cardiol..

[59] Bonilla I.M., Sridhar A., Györke S., Cardounel A.J., Carnes C.A. (2012). Nitric oxide synthases and atrial fibrillation. Front. Physiol..

[60] Santos-Miranda A., Joviano-Santos J.V., Ribeiro G.A., Botelho A.F.M., Rocha P., Vieira L.Q., Cruz J.S., Roman-Campos D. (2020). Reactive oxygen species and nitric oxide imbalances lead to in vivo and in vitro arrhythmogenic phenotype in acute phase of experimental Chagas disease. PLoS Pathog..

[61] Paapstel K., Kals J., Eha J., Tootsi K., Ottas A., Piir A., Jakobson M., Lieberg J., Zilmer M. (2018). Inverse relations of serum phosphatidylcholines and lysophosphatidylcholines with vascular damage and heart rate in patients with atherosclerosis. Nutr. Metab. Cardiovasc. Dis..

[62] Wirleitner B., Rudzite V., Neurauter G., Murr C., Kalnins U., Erglis A., Trusinskis K., Fuchs D. (2003). Immune activation and degradation of tryptophan in coronary heart disease. Eur. J. Clin. Invest.

[63] Yu E., Ruiz-Canela M., Guasch-Ferré M., Zheng Y., Toledo E., Clish C.B., Salas-Salvadó J., Liang L., Wang D.D., Corella D. (2017). Increases in Plasma Tryptophan Are Inversely Associated with Incident Cardiovascular Disease in the Prevención con Dieta Mediterránea (PREDIMED) Study. J. Nutr..

[64] Gostner J.M., Kurz K., Fuchs D. (2020). The significance of tryptophan metabolism and vitamin B-6 status in cardiovascular disease. Am. J. Clin. Nutr..

[65] Murr C., Grammer T.B., Kleber M.E., Meinitzer A., März W., Fuchs D. (2015). Low serum tryptophan predicts higher mortality in cardiovascular disease. Eur. J. Clin. Investig..

[66] Kwiatkowska I., Hermanowicz J.M., Mysliwiec M., Pawlak D. (2020). Oxidative Storm Induced by Tryptophan Metabolites: Missing Link between Atherosclerosis and Chronic Kidney Disease. Oxidative Med. Cell. Longev..

[67] Yang L., Wang L., Deng Y., Sun L., Lou B., Yuan Z., Wu Y., Zhou B., Liu J., She J. (2020). Serum lipids profiling perturbances in patients with ischemic heart disease and ischemic cardiomyopathy. Lipids Health Dis..

[68] Tan S.T., Ramesh T., Toh X.R., Nguyen L.N. (2020). Emerging roles of lysophospholipids in health and disease. Prog. Lipid Res..

[69] Aitken-Buck H.M., Krause J., Zeller T., Jones P.P., Lamberts R.R. (2020). Long-Chain Acylcarnitines and Cardiac Excitation-Contraction Coupling: Links to Arrhythmias. Front. Physiol..

[70] Zhao S., Feng X.F., Huang T., Luo H.H., Chen J.X., Zeng J., Gu M., Li J., Sun X.Y., Sun D. (2020). The Association Between Acylcarnitine Metabolites and Cardiovascular Disease in Chinese Patients With Type 2 Diabetes Mellitus. Front. Endocrinol..

[71] Volani C., Paglia G., Smarason S.V., Pramstaller P.P., Demetz E., Pfeifhofer-Obermair C., Weiss G. (2018). Metabolic Signature of Dietary Iron Overload in a Mouse Model. Cells.

[72] Ferrari R., Merli E., Cicchitelli G., Mele D., Fucili A., Ceconi C. (2004). Therapeutic effects of L-carnitine and propionyl-L-carnitine on cardiovascular diseases: A review. Ann. N. Y. Acad. Sci..

[73] Gao X., Zhang W., Wang Y., Pedram P., Cahill F., Zhai G., Randell E., Gulliver W., Sun G. (2016). Serum metabolic biomarkers distinguish metabolically healthy peripherally obese from unhealthy centrally obese individuals. Nutr. Metab..

[74] Xu W.Y., Shen Y., Zhu H., Gao J., Zhang C., Tang L., Lu S.Y., Shen C.L., Zhang H.X., Li Z. (2019). 2-Aminoadipic acid protects against obesity and diabetes. J. Endocrinol..

[75] Mangge H., Stelzer I., Reininghaus E.Z., Weghuber D., Postolache T.T., Fuchs D. (2014). Disturbed tryptophan metabolism in cardiovascular disease. Curr. Med. Chem..

[76] Tomczyk M.M., Dolinsky V.W. (2020). The Cardiac Lipidome in Models of Cardiovascular Disease. Metabolites.

[77] Smyth G.K. (2004). Linear models and empirical bayes methods for assessing differential expression in microarray experiments. Stat. Appl. Genet. Mol. Biol..

[78] Jewison T., Su Y., Disfany F.M., Liang Y., Knox C., Maciejewski A., Poelzer J., Huynh J., Zhou Y., Arndt D. (2014). SMPDB 2.0: Big improvements to the Small Molecule Pathway Database. Nucleic Acids Res..

[79] Xia J., Wishart D.S. (2016). Using MetaboAnalyst 3.0 for Comprehensive Metabolomics Data Analysis. Curr. Protoc. Bioinform..

